# Duration of Mechanical Ventilation in the Emergency Department

**DOI:** 10.5811/westjem.2017.5.34099

**Published:** 2017-07-11

**Authors:** Lauren B. Angotti, Jeremy B. Richards, Daniel F. Fisher, Jeffrey D. Sankoff, Todd A. Seigel, Haitham S. Al Ashry, Susan R. Wilcox

**Affiliations:** * Medical University of South Carolina, Division of Pulmonary, Critical Care, Allergy and Sleep Medicine, Charleston, South Carolina; † Massachusetts General Hospital, Respiratory Care Services, Boston, Massachusetts; ‡ University of Colorado at Denver, School of Medicine, Department of Emergency Medicine, Denver, Colorado; § Kaiser Permanente East Bay, Oakland and Richmond Medical Centers, Department of Emergency Medicine and Critical Care, Oakland, California; ¶ Medical University of South Carolina, Division of Emergency Medicine, Charleston, South Carolina

## Abstract

**Introduction:**

Due to hospital crowding, mechanically ventilated patients are increasingly spending hours boarding in emergency departments (ED) before intensive care unit (ICU) admission. This study aims to evaluate the association between time ventilated in the ED and in-hospital mortality, duration of mechanical ventilation, ICU and hospital length of stay (LOS).

**Methods:**

This was a multi-center, prospective, observational study of patients ventilated in the ED, conducted at three academic Level I Trauma Centers from July 2011 to March 2013. All consecutive adult patients on invasive mechanical ventilation were eligible for enrollment. We performed a Cox regression to assess for a mortality effect for mechanically ventilated patients with each hour of increasing LOS in the ED and multivariable regression analyses to assess for independently significant contributors to in-hospital mortality. Our primary outcome was in-hospital mortality, with secondary outcomes of ventilator days, ICU LOS and hospital LOS. We further commented on use of lung protective ventilation and frequency of ventilator changes made in this cohort.

**Results:**

We enrolled 535 patients, of whom 525 met all inclusion criteria. Altered mental status without respiratory pathology was the most common reason for intubation, followed by trauma and respiratory failure. Using iterated Cox regression, a mortality effect occurred at ED time of mechanical ventilation > 7 hours, and the longer ED stay was also associated with a longer total duration of intubation. However, adjusted multivariable regression analysis demonstrated only older age and admission to the neurosciences ICU as independently associated with increased mortality. Of interest, only 23.8% of patients ventilated in the ED for over seven hours had changes made to their ventilator.

**Conclusion:**

In a prospective observational study of patients mechanically ventilated in the ED, there was a significant mortality benefit to expedited transfer of patients into an appropriate ICU setting.

## INTRODUCTION

Hospital crowding, leading to boarding patients in the emergency department (ED), is a common problem nationwide with crowding reported in 90% of EDs, 40% of which report crowding on a daily basis.^1^ Boarding is a particular problem for patients awaiting intensive care unit (ICU) beds; the American Hospital Association (AHA) reports an average ED boarding time of six hours for critically ill patients in crowded EDs.^2^ Multiple studies worldwide have illuminated the detrimental effect of ED crowding on patient outcomes and mortality.^2^–^8^ Delay in transfer of mechanically ventilated patients from the ED to the ICU has been associated with higher in-patient mortality and longer hospital length of stay (LOS).^2^,^8^,^9^

With the aging population and advances in care of chronic medical conditions, ED crowding and the need to manage critically ill patients in the ED will continue to increase. Previously, urban EDs have been shown to provide up to 150 days of critical care time per year, and this trend is increasing.^1^,^10^ One prior retrospective review of a national database of ED visits found ED LOS for critically ill patients has been increasing by 7% per year.^11^ ED staffing and organization are generally not conducive to delivering the personalized care critically ill patients require. Emergency physicians (EP) have limited time for ongoing management of critically ill patients, and ED nurses are rarely staffed at the 1:1 or 1:2 nurse-to-patient ratio common in most ICU settings. Additionally, the population of patients needing prolonged acute mechanical ventilation (defined as >96 hours) is projected to grow at a rate of 5.5% per year. 12

Although the first hours of management in a critically ill patient can be pivotal in terms of outcome,^13^–^16^ many patients in the ED, including those with acute respiratory distress syndrome (ARDS), are not ventilated with lung-protective ventilation,^17^–^19^ and the majority of patients have no changes made to their ventilators while in the ED.^18^,^19^ Every hour of additional mechanical ventilation in the ED has been associated with a 20% increased risk of developing pneumonia in blunt trauma patients.^20^ Therefore, we performed a prospective, observational study of mechanically ventilated patients boarding in the ED, awaiting admission to an ICU bed. We hypothesized that those patients with a longer duration of mechanical ventilation in the ED would have increased in-hospital mortality, longer duration of mechanical ventilation, and longer ICU and hospital LOS.

## METHODS

This was a multi-center, prospective, observational cohort study of patients ventilated in the ED, conducted at three academic emergency departments in the United States from July 2011 to March 2013. All three EDs are Level I Trauma Centers with over 100,000 ED visits a year, staffed with board-certified EPs and emergency medicine residents. All consecutive adult patients on invasive mechanical ventilation via an endotracheal tube or tracheostomy tube were eligible for enrollment. Exclusion criteria included death upon arrival or during ED course, or direct transfer to the operating room (OR) from the ED. We also excluded patients who did not have complete documentation regarding the duration of time ventilated in the ED and ED LOS.

Population Health Research Capsule

What do we already know about this issue?

Extended boarding in the emergency department (ED) has been associated with increased morbidity and mortality.

What was the research question?

Is there an association between duration of ventilation in mechanically ventilated patients boarding in the ED with mortality?

What was the major finding of the study?

Older patient age and intubation for neurologic issues were independently associated with increased mortality.

How does this improve population health?

Triaging high-risk patients for transfer to the ICU and increased attention to ventilator management in the ED may improve patient outcomes.

Patients were screened and enrolled upon presentation to the ED while receiving invasive mechanical ventilation or after intubation in the ED. Patients were prospectively screened by trained research assistants (RAs) seeking patients receiving invasive mechanical ventilation on presentation to the ED or after intubation in the ED at each of the three study sites. RAs then enrolled the patients presenting during the hours of RA presence in the ED, collecting all data regarding demographics, indication for intubation and ventilation, initial ventilator settings, any changes made to ventilator settings, and blood gas data. RAs also collected data from the remainder of the hospitalization for each enrolled patient, including ventilator days, ICU LOS, hospital LOS, and mortality. RAs worked closely with respiratory therapists at each center to collect all ventilator settings and changes. Data monitoring was performed by each site’s local primary investigator. This study was funded in part by a university development grant, and the study duration and sample size was determined by convenience sampling during the grant funding period.

To assess the effects of duration of mechanical ventilation in the ED, rather than entire ED LOS, we defined the time ventilated in the ED as the time of presentation to the ED for those initiated on ventilation prior to arrival, or the time of intubation, for the remainder of patients, until the time of ICU admission. Patients were classified by the indication for intubation including altered mental status with no overt respiratory pathology, trauma, cardiac arrest, respiratory failure, neurologic events, and other causes. We defined subgroups of interest by the most common indications for intubation, including altered mental status, trauma, and respiratory failure. We included any recorded modification of ventilator settings as a change in settings, from changing the mode to decreasing the fraction of inspired oxygen. Lung protective ventilation was defined as a tidal volume of 8mL/kg or less of predicted body weight, with full details published previously.^19^ (See Appendix A.)

The time of intubation, time of transfer to an ICU, admitting ICU, duration of mechanical ventilation, ICU LOS, hospital LOS, and in-hospital mortality were recorded. Our primary outcome was in-hospital mortality, with secondary outcomes of ventilator days, ICU and hospital LOS. To reduce the risk of survivor bias, we excluded patients who died from the secondary outcome analyses.

Institutional review boards for all participating institutions approved the study protocols with waiver for informed consent.

Data were input into Microsoft Excel (Microsoft Corp., Redmond, WA) and then transferred to SPSS (version 21.0, IBM Corp, Armonk, NY) for statistical analysis. We visually inspected data and excluded missing data on a case-by-case basis. The effect of duration of mechanical ventilation in the ED on in-hospital mortality was analyzed by univariate Cox regression analysis. Specifically, we assessed a significant effect of duration of mechanical ventilation in the ED on mortality via iterative analyses using hour-based time points, such as <4 hours, <5 hours, in a stepwise fashion.

We performed descriptive analyses of relevant clinical outcomes for the entire cohort, as well as for patients ventilated in the ED for less than and more than seven hours. Continuous variables are reported as means and standard deviations (SD), and categorical variables are reported as numbers and percentages. The frequency of ventilator changes made among subgroups classified by indication for intubation was compared by chi-squared analyses. We assessed differences between continuous variables using single-factor ANOVA, while categorical variables were determined by chi-square testing or two-sided Student’s T test with unequal variance as appropriate. Two-tailed Pearson’s correlations were performed to assess for simple associations between clinical parameters and outcomes of interest. We performed multivariable regression analyses to assess for independent associations between clinical and patient parameters and mortality. An alpha of less than 0.05 was considered statistically significant for all analyses.

## RESULTS

We enrolled 535 patients. Ten were excluded as their times in the ED were not fully documented, leaving 525 patients for final analysis (n=525). Sixty percent of patients were male and the average age was 55.6 years (range 18 to 96 years) (Table 1). Sixty-one percent of patients were intubated in the ED, with the remaining 39% intubated prior to arrival. Altered mental status without respiratory pathology was the most common reason for intubation (38.3%), followed by trauma (23.2%) and respiratory failure (17.1%). The primary disposition for patients was a medical ICU (52.7%), with 23.7% being admitted to a surgical/trauma ICU (STICU), and 16.3% to a neurosciences ICU. The mean duration of mechanical ventilation in the ED in this cohort was 4 hours and 28 minutes, with SD of 4 hours and 18 minutes.

Univariate Cox regression analysis demonstrated a significant increase in mortality with duration of mechanical ventilation for all time points of more than seven hours of mechanical ventilation in the ED. The hazard ratio (HR) for mortality for >7 hours of mechanical ventilation in the ED was 1.31 (95% confidence intervals [CI] [1.03–1.70], P < 0.001), and the HR remained significant for all time points greater than seven hours (Figure).

Of the 525 patients enrolled, 461 were ventilated in the ED for less than seven hours, and 64 were ventilated in the ED for greater than seven hours (Table 1). The cohort of patients ventilated for less than seven hours was younger and more likely to be ventilated for cardiac arrest or airway edema, although the numbers of patients intubated for these indications were small, with 34 total for cardiac arrest and 12 with airway edema (Table 1).

Patients in the greater-than-seven-hour group were more likely to receive initial lung protective ventilation, yet they were less likely to have any changes made to their ventilator during their time in the ED. More patients in the less-than-seven-hour group were admitted to the STICU, and more patients in the greater-than-seven-hour group were admitted to the neuro ICU.

Patients who remained ventilated in the ED greater than seven hours had significantly higher in-hospital mortality at 45.9% versus 29.4% (p=0.018) for those who were ventilated in the ED for less than seven hours (Table 2).

The greater-than-seven-hour group also had a longer duration of mechanical ventilation, at 4.8 days compared to 2.5 days, (p=0.011). ICU LOS and hospital LOS did not differ significantly between the two groups.

The frequency of lung protective ventilation was not significantly different between any of the subgroups, including patients intubated for altered mental status vs. respiratory failure (P=0.22), trauma vs. respiratory failure (P=0.14), or altered mental status vs. trauma (P=0.66). Both the subgroups of patients intubated for altered mental status and those intubated for trauma had a higher rate of ventilator changes in the ED compared to those intubated for respiratory failure (28.4% versus 13.3%, P=0.002 and 25.4% versus 13.3%, P=0.03, respectively). There was no statistically significant difference between altered mental status and trauma patients (P=0.56).

For the subgroup of patients intubated for altered mental status, those patients ventilated in the ED > 7 hours were associated with an overall longer duration of ventilation, at 5.81 to 1.5 days, (p=0.05) (Table 3).

The extended duration of ventilation in the ED of over seven hours was also associated with significantly increased mortality in trauma patients (43.8% vs. 15.2%, P= 0.046) and patients with respiratory failure (72.7% vs. 32.9%, P=0.02).

Age, use of lung protective ventilation, changes made to ventilator settings in the ED, admission to the neurosciences ICU, admission to the STICU, and duration of mechanical ventilation were assessed as independent variables for their effect on in-hospital mortality. As the intubation for cardiac arrest group had only one patient in the greater-than-seven-hour cohort, and there were no patients who remained in the ED for greater than seven hours for airway edema, these factors were excluded from further analysis. Multivariate analysis demonstrated that age and admission to the neurosciences ICU, with an odds ratio of 2.210 (95% CI 1.286–3.800, P= 0.004) were independently associated with mortality (Table 4).

Bivariate two-tailed Pearson correlations demonstrated moderate positive correlation for death and age (ρ = 0.33, P<0.001) and weak correlation for death and admission to the neurosciences ICU (ρ = 0.18, P<0.001). Weak but significant negative correlations were determined for death and admission to the STICU (ρ = −0.14, P = 0.002) and mechanical ventilation of >7 hours in the ED (ρ = −0.12, P = 0.009). All other correlations were not significant.

## DISCUSSION

This study is the first prospective, multi-center, observational study assessing outcomes associated with duration of mechanical ventilation in the ED. The increased mortality correlated with a duration of mechanical ventilation in the ED of over seven hours in this cohort is consistent with prior retrospective studies^21^ and recommended quality benchmarks,^8^ including those focused on critically ill or ventilated patients, finding that an ED LOS over six hours is associated with worse outcomes. A retrospective cross-sectional analysis of the IMPACT database conducted by Chalfin et al., found both increased mortality and increased hospital LOS in critically ill ED patients whose transfer to the ICU was delayed over six hours.^2^ Similarly, Hung and colleagues found that a greater-than-four-hour ED LOS for mechanically ventilated patients increased the 21-day mortality in their single center, retrospective cohort.^8^ The importance of these findings is put into perspective when considering that the AHA reports a mean wait of six hours for an ICU bed in crowded EDs,^2^ and this is supported by other studies.^22^ The ED LOS in this study was similar to these reports, over five hours, with a mean duration of ventilation of over 4.5 hours. A minority of patients, approximately one in eight, were ventilated for over seven hours in the ED.

The two groups in this study were not equivalent, as patients waiting in the ED for over seven hours were older and were more likely to be admitted to the neurosciences ICU, while the less-than-seven-hour group included more patients admitted to the STICUs. In multivariate analysis, only older age and admission to the neurosciences ICU were independently associated with increased mortality. These results demonstrate that while increased ED boarding time is a confounder for mortality, boarding time was not independently significantly associated with mortality in this cohort. Increased ED boarding time may have effects in a broader population, however, and future studies assessing the role of boarding time as a contributor to or confounder of mortality are necessary.

However, the observation that patients with neurologic emergencies and those who were older were more likely to board in the ED while ventilated, while younger patients and those admitted to the STICU had shorter ED ventilation times, is an important finding. Patients with neurologic injuries require close monitoring of mechanical ventilation and hemodynamics, and multiple studies have shown that these patients have a significantly lower mortality rate when cared for in a dedicated neurocritical care unit.^23^,^24^ Additionally, older age has been independently associated with increased mortality in the ICU.^25^,^26^ Therefore, the findings of this investigation support the importance of transferring ventilated patients with neurologic injury and older patients to the ICU as soon as possible.

We previously reported that despite prolonged duration of ventilation in the ED, only 22.2% of patients in a subgroup of this cohort had any ventilator changes made in the ED, with the majority of those changes being adjustments to the respiratory rate and FiO2.^19^ Of patients initially ventilated without lung protective ventilation, only 7% were changed to lung protective settings in the ED. These results, consistent with prior studies of ventilation in the ED,^18^ suggest that once ventilator settings are selected in the ED, adjustments to the ventilator are infrequent and often trivial. One may anticipate that those patients who board the longest would be more likely to have changes made to their ventilator while waiting in the ED, but our findings were the converse. Twice as many patients in the less-than-seven-hour group had ventilator changes as compared to the greater-than-seven-hour group, despite the prolonged ED boarding time. Interestingly, the subgroups intubated for altered mental status and trauma were also more likely to have changes made to their ventilators as compared to those intubated for respiratory failure. Yet in our cohort, patients intubated with respiratory failure who ventilated in the ED for over seven hours had a mortality rate of approximately 73%, compared to 33% for those ventilated less than seven hours.

Emergency medicine residents^27^ and EPs^28^ have expressed relative discomfort with management of mechanical ventilation, and the majority surveyed cede responsibility for ventilator management to respiratory therapists.^27^,^28^ Whether these factors, especially in patients with respiratory failure or neurocritical care patients who require close monitoring, account for the observed increase in mortality is unknown.

Numerous hospital and healthcare system factors may impact ED LOS,^29^ and these factors may also impact the care provided to patients boarding in the ED. Although EDs have seen consistent increases in volume and patient acuity,^30^–^34^ the number of ED beds and acute-care hospital beds have declined over the last two decades,^35^,^36^ leading to more boarding of ever higher acuity patients. Intensivist and ICU nursing shortages hinder efficient transfer of patients to ICUs and prohibit early intensivist involvement in the care of critically ill patients. A recent study found that ICU crowding, with ICUs functioning at greater than 20% above the average annual census, was associated with an increased ED LOS.^29^ With these dual factors of increasing acuity with worsening crowding, the incidence of mechanically ventilated patients in the ED is growing^37^ and their LOS in the ED is increasing.^22^ EPs, therefore, may be primarily responsible for prolonged management of mechanically ventilated patients.^22^,^32^,^33^ Future efforts should jointly focus on increasing EPs’ knowledge of and comfort with managing ventilated patients, while simultaneously working to remove barriers for expeditious ICU admission.

The creation of an ED-based ventilator care bundle, as proposed by Easter and colleagues,^9^ may impact mortality and morbidity in this cohort with widespread implementation. A ventilator care bundle could be automated after intubation in the ED and could include such measures as elevation of head of bed, an arterial blood gas within 30 minutes of intubation and post-intubation chest radiography. A randomized trial comparing implementation of standardized post-intubation care to routine care in the ED would be of great interest. Notably, Fuller and colleagues recently published results of a quasi-experimental trial using an ED ventilator protocol for patients with ARDS finding their protocol to be feasible and associated with increased ventilator-free days and decreased mortality.^38^

## LIMITATIONS

As an observational study, our findings have several limitations. Additionally, only correlative associations could be made while causal relationships could not be determined. Multiple confounding factors may have significantly impacted the results, and the effect of confounders could not be determined based on the available data. We did not have ASA scoring or APACHE scores for this cohort to compare severity of illness between the groups. Triage decisions may have impacted the outcomes, as patients with potentially reversible causes of critical illness may have been dispositioned more rapidly to receive definitive care. Our data reflect a greater proportion of patients with neurologic conditions in the > 7 hour group, possibly signifying a perceived unfavorable prognosis at the onset. Nearly 40% of our patients were intubated prior to ED arrival. Although ED transport time is minimal in urban settings,^39^ this may have confounded our data set. Due to limitations in funding, these patients represent a convenience sample, and this sampling may have impacted the results.

## CONCLUSION

In this cohort, there was a significant reduction in mortality and the total duration of mechanical ventilation associated with duration of mechanical ventilation in the ED of less than seven hours, although there were no differences in ICU or hospital LOS. Older age and admission to the neurosciences ICU were independently associated with increased mortality. Few patients had changes to their ventilator settings while boarding in the ED, and those who waited the longest were actually least likely to have any changes made. Although these patients may benefit most from prompt transfer to an ICU, crowding and limited resources currently limit this option. Therefore, the creation of a ventilator care bundle in the ED, with increased attention to ventilator management, may be a feasible way to impact patient outcomes.

## Supplementary Information



## Figures and Tables

**Figure:**
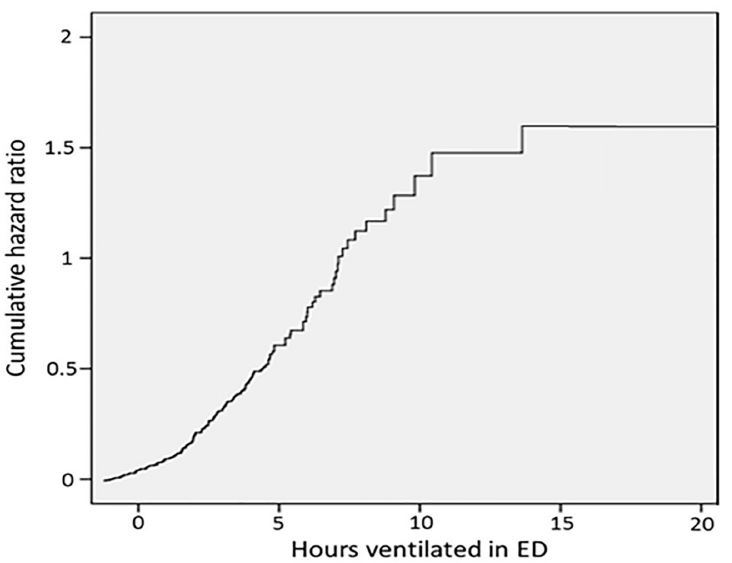
The study sample size and distribution of duration of mechanical ventilation in the ED were insufficiently powered to perform Cox regression for time points of less than four hours.

**Table 1:** Demographics of patients enrolled in a study of the association between duration of mechanical ventilation in the emergency department and in-hospital mortality.

Variable	Total (n=525)	Less than 7 hours (n=461)	More than 7 hours (n=64)	p value
Male patients n (%)	313 (59.6)	308 (66.9)	36 (56.3)	0.922
Mean age (years [IQR])	55.6 [41.7–69.2]	54.6 [41.5–67.8]	63.5 [50.2–78.4]	<0.001
Patients intubated in the ED n (%)	320 (60.9)	281 (61.0)	37 (57.8)	0.646
Indication for intubation n (%)				
AMS	201 (38.3)	179 (38.8)	22 (34.4)	0.480
Trauma	122 (23.2)	104 (22.6)	18 (28.1)	0.360
Respiratory failure	90 (17.1)	79 (17.1)	11 (17.2)	0.998
ICH or seizure	51 (9.7)	41 (8.9)	10 (15.6)	0.163
Cardiac arrest	34 (6.5)	33 (7.2)	1 (1.6)	0.005
Airway edema	12 (2.3)	12 (2.6)	0 (0)	<0.001
Other	15 (2.9)	13 (2.8)	2 (3.1)	0.824
Management of ventilation in the ED				
Lung protective ventilation n (%)	345(65.8)	296 (64.3)	49 (76.2)	0.047
Any ventilator changes while in ED n (%)	115 (21.9)	107 (23.2)	8 (12.5)	0.022
Disposition n (%)				
Medical ICU	277 (52.7)	245 (53.2)	31 (49.2)	0.559
Surgical trauma ICU	124 (23.7)	115 (24.9)	9 (14.3)	0.032
Neuro ICU	86 (16.3)	66 (14.4)	19 (30.2)	0.011
Cardiovascular ICU	38 (7.3)	34 (7.4)	4 (6.3)	0.744

*ED,* emergency department; *ICU*, intensive care unit; *IQR*, interquartile range; *AMS*, altered mental status; *ICH*, intracranial hemorrhage.

**Table 2:** Outcomes for patients mechanically ventilated in the ED for greater or less than seven hours.

Time ventilated in the ED	< 7 hours	> 7 hours	p value
Mortality (%)	29.4	45.9	0.018
Duration of mechanical ventilation (days)	2.5	4.8	0.011
ICU length of stay (days)	5.2	7.2	0.227
Hospital length of stay (days)	14.0	14.9	0.831

*ED,* emergency department; *ICU*, intensive care unit.

**Table 3:** Outcome sub-group analyses.

Subgroup variables	< 7 hours	> 7 hours	p value
Altered mental status			
Lung protective ventilation (%)	62.6	90.9	<0.001
Any ventilator changes while in ED (%)	30.7	9.1	0.005
Mortality (%)	26.0	30.0	0.711
Mechanical ventilation duration (days)	1.5	5.81	0.050
ICU length of stay (days)	3.79	7.40	0.442
Hospital length of stay (days)	10.65	13.58	0.431
Trauma			
Lung protective ventilation (%)	68.3	66.7	0.898
Any ventilator changes while in ED (%)	26.0	22.2	0.736
Mortality (%)	15.2	43.8	0.046
Mechanical ventilation duration (days)	3.01	4.52	0.714
ICU length of stay (days)	6.98	6.77	0.972
Hospital length of stay (days)	16.0	8.7	0.039
Respiratory failure			
Lung protective ventilation (%)	55.8	72.7	0.286
Any ventilator changes while in ED (%)	13.9	9.1	0.633
Mortality (%)	32.9	72.7	0.020
Mechanical ventilation duration (days)	3.29	2.70	0.880
ICU length of stay (days)	5.22	11.1	0.104
Hospital length of stay (days)	15.5	26.8	0.305

*ED,* emergency department; *ICU*, intensive care unit.

**Table 4:** Multivariable regression analysis demonstrating association between age and admission to the neurosciences ICU.

Variable	Odds ratio for mortality (95% confidence intervals)	p value
Age	0.962 (0.950–0.974)	<0.001
Use of lung protective ventilation	0.860 (0.554–1.334)	0.500
Ventilator changes in the ED	1.036 (0.608–1.765)	0.896
Admission to Neuro ICU	2.210 (1.286–3.800)	0.004
Admission to the STICU	0.837 (0.475–1.476)	0.539
Duration of mechanical ventilation (>7 hours or <7 hours)	1.463 (0.796–2.690)	0.221

*ED,* emergency department; *ICU*, intensive care unit.
