# Supplementary material for: Duration of Mechanical Ventilation in the Emergency Department
Source: West J Emerg Med. 2017 Jul 11;18(5):972–9. doi: 10.5811/westjem.2017.5.34099 (PMC5576636; doi:10.5811/westjem.2017.5.34099)
Supplement: Supplementary file 1 [file wjem-18-972-s001.docx]

Appendix A

Patients’ heights were measured and recorded on presentation. Predicted body weight was calculated as 50 kg + 2.3 kg for each inch in height over five feet for male patients, and 45.5 kg + 2.3 kg for each inch over five feet for female patients. For both volume and pressure-controlled settings, we calculated tidal volume in mL/kg by dividing the tidal volume by predicted body weight. Lung protective ventilation was defined as a tidal volume of 8mL/kg or less. For patients who had a plateau pressure checked in the ED, if the value was > 30cmH2O, even for tidal volumes less than or equal to 8mL/kg, we classified the ventilation as being non-lung protective.
